# Cancer chemoprevention: lessons learned and future directions

**DOI:** 10.1038/sj.bjc.6602765

**Published:** 2005-09-06

**Authors:** D E Brenner, A J Gescher

**Affiliations:** 1 Division of Hematology-Oncology, Department of Internal Medicine, Department of Pharmacology, University of Michigan & VA Medical Center, Ann Arbor, MI 48109, 0930, USA; 2 Cancer Biomarkers and Prevention Group, Department of Cancer Studies, University of Leicester, Leicester LE2 2LX, UK

**Keywords:** chemoprevention, neoplasia, biomarkers, nutrition, drug therapy

## Abstract

The concept of delaying or preventing epithelial transformation remains a viable and attainable goal for the future. Drug-based strategies for chemoprevention of the future may predominantly rely upon targeted therapies with tolerable but defined toxicities for treatment of individuals diagnosed with intraepithelial neoplasias. Foods, diet manipulation strategies, or nutraceuticals may be more appropriate to delay or prevent carcinogenesis progression in healthy populations with genetic or epidemiologic evidence of risk for future transformation.

Three recent publications have demonstrated an unacceptable therapeutic index due to cardiovascular toxicity of selective cyclooxygenase-2 inhibitors when used for cancer preventive or anti-inflammatory indications (Bresalier *et al*, 2005; Nussmeier *et al*, 2005; Solomon *et al*, 2005). These data have focused the debate on whether pharmacologic interventions aimed at delaying or preventing the transformation of organ epithelia should proceed. Further, bad news for proponents of the cancer chemoprevention approach emerged recently from a trial of *α*-tocopherol supplements in patients with stage I or II head and neck cancer treated by radiation therapy (Bairati *et al*, 2005). In these patients, the incidence of second primary cancers was higher (hazard ratio: 2.88) than that in patients who received the placebo. The corollaries of these clinical results have been amply debated (for example, see Alberts *et al*, 2005; Drazen, 2005; Meyskens and Szabo, 2005). In this minireview, we wish to distill some crucial issues from that debate, and re-evaluate current and prospective chemoprevention strategies considered promising in the light of these issues.

The recent cancer chemoprevention trial experiences have directed the focus on the following four pivotal issues:

(1) *Acceptable therapeutic index for interventions in healthy populations*: Individuals who are treated for a full-blown malignancy accept toxic therapies as a price for improved quality of life, survival, or both. In contrast, the acceptable therapeutic index for interventions intended for healthy individuals at risk of cancer in the future has ‘… been both underestimated and overestimated from failure to critically assess the parameter in relation to the modulation of the relevant biologic/biochemical/molecular end-points’ (Meyskens and Szabo, 2005).

In the case of cardiovascular disease prevention, the individuals and physicians accept treatment-induced toxicity if the recipient is convinced that a ‘disease’ such as hypertension or hvpercholesterolemia is being treated. In the case of neoplastic risk, healthy individuals at high risk for future development of a malignancy, for example, individuals with highly penetrant, low-frequency mutations (e.g. BRCA, familial adenomatous polyposis), are usually willing to tolerate surgical procedures such as colectomy, mastectomy, and oophorectomy to reduce cancer risk. Irritating, yet minor, adverse effects (e.g. hypokalemia associated with diuretics, headaches) are less acceptable for intervention in healthy populations, who are not ‘diagnosed’ with clear and imminent ‘disease’, but rather are approached to undergo an intervention on the basis of a conceptually abstract ‘epidemiologic risk profile’. Major toxicities such as the recently reported enhanced risk of cardiovascular events associated with the administration of cyclooxygenase-2 inhibitors are unacceptable for any intervention with chemopreventive intent. While these examples represent opposite ends of a continuum of risk–benefit for chemopreventive intervention, risk–benefit for the bulk of individuals at risk for sporadic neoplasms is less clear and poorly defined.

(2) *Existence of preliminary data to rationalise trials in humans*: Epidemiologic data, that is, hypotheses-generating associations, have not accurately predicted prospective cancer preventive outcomes to date with sufficient certainty to justify large-scale, high-cost trials with a cancer end-point (Meyskens and Szabo, 2005). Rodent models of carcinogenesis, either transgenic or chemically induced, provide tools to probe carcinogenesis mechanisms and to obtain preliminary evidence of potential preventive efficacy (Hawk *et al*, 2005). Nevertheless, the knowledge accrued thus far in such models suggests that their usefulness to predict therapeutic indices of cancer chemopreventive agents is rather doubtful (Corpet and Pierre, 2003).

(3) *Trial design and progression of research in humans*: The mixed record of success of the development of novel chemical entities by the pharmaceutical industry as cancer chemopreventive drugs points to the importance of design, implementation, and interpretation of phase I and II research in humans. An optimal cancer preventive dose cannot be extrapolated from doses used for different therapeutic indications. For example, in the chemoprevention of colorectal adenocarcinomas, higher doses of aspirin may be less effective than lower ones, which also protect against cardiovascular disease (Baron *et al*, 2003). Recent discussions have emphasised the importance of studying fundamental dose–response relationships in the field of cancer chemoprevention followed by extended biomarker-driven phase II efficacy trials in humans prior to embarking upon large, long, costly phase III risk reduction trials with cancer end-points (Alberts *et al*, 2005; Meyskens and Szabo, 2005).

*(4) Importance of biomarkers as potential surrogates for preventive efficacy*: Meyskens and Szabo (2005) cogently described the conceptual, syntactic, and scientific confusion currently surrounding biomarkers as potential chemoprevention end-points. Nevertheless, future definition of therapeutic efficacy of interventions designed to delay or prevent neoplastic progression requires discovery and validation of genetic or biochemical processes that reliably reflect the future potential of target epithelia to progress towards transformation. Current efforts by the US National Cancer Institute (NCI) to support and systematise biomarker discovery, validation, and application through the Early Detection Research Network (EDRN) (Verma and Srivastava, 2003) and Specialized Organ Research Effort (SPORE) mechanisms provide the necessary complex infrastructure. While splitting the development and validation of biomarkers from measurement of efficacy of preventive intervention reduces the rate of progress during the short term, such efforts will enhance the quality and speed of translational progress of new intervention products for preventive indications in the long run.

One might conclude that efforts to develop new chemopreventive interventions should be de-emphasised, and that resources should be redirected to more fundamental research. The experience gained in cancer chemoprevention trials to date provides important lessons that suggest a more rational, scientifically driven mode of development than that which has been often pursued until now. This process should enable a systematic, stepwise, phased exploration in preclinical models and in humans of newly emerging interventions. In the light of these considerations, we discuss below a chemopreventive agent selection strategy of novel drugs and a number of specific agents with putative cancer chemopreventive potential, of which some are currently under active clinical investigation and others originate from novel sources still at the stage of preclinical development.

## PREVENTIVE AGENT SELECTION FOR DEVELOPMENT IN HUMANS

The recent experiences gained in the development of drugs as cancer chemopreventive agents lead to the conclusion that the therapeutic index of proposed interventions should be titrated to relative risk of transformation. Individuals at high risk of developing malignancies with proven pathological evidence of potential transformation (‘intraepithelial neoplasia’), for example individuals with adenomatous polyps, atypical ductal hyperplasia, or oral leukoplakia, might be considered suitable candidates for ‘treatment of their disease’ using interventions consisting of single or combined drug-based approaches. Optimally, such drugs should target specific molecular carcinogenesis profiles obtained via genetic or proteomic profiling of that individual's pathologic lesion. Drugs that target intracellular regulation of proliferation, survival, and angiogenesis may be suitable to be used in such individuals with preneoplasias as compared to those who suffer from advanced full-blown malignancies (i.e. transformed, invasive cancers). Such an approach might start with small, rapidly implemented and brief (60 days or less) translational clinical trials of biomarker modulation by multiple doses of a given intervention, alone or in combination with other mechanism-based agents. Subsequent biomarker-driven trials of drug combinations require duration of a year or more to assess both toxicity and continued biomarker effects prior to consideration of large, population-based cancer end-point trials. Toxicity profiles should include quality of life instruments to reflect the potential adverse nature of such interventions (Stanton *et al*, 2005).

Strategies for the prevention of malignancies in patients at higher than normal risk of developing cancer but without evidence of pathologic progression can exploit concepts established in the treatment of cardiovascular risk factors. Hypertension and hypercholesterolaemia are considered ‘diseases’ that are treated by physicians despite the fact that these end-points are biomarkers for future cardiovascular events. Critical to the future implementation of cancer preventive interventions using drugs are the ability to alter public opinion and to prompt primary medical care providers to adjust their perception concerning high-risk cancer biomarkers – in analogy to treatment of cardiovascular biomarkers – and willingness to accept mild toxicity. For such individuals, a nutrition-based approach might be more acceptable and potentially efficacious.

## PROMISING DRUG-BASED CHEMOPREVENTION APPROACHES

### Nonsteroidal anti-inflammatory agents (NSAIDs)

In the wake of the recent observations of unacceptable cardiovascular effects of selective cyclooxygenase-2 inhibitors, and mindful of the long-known gastro-intestinal irritation/damage associated with long-term consumption of NSAIDs, is this class of agent still viable as potential cancer chemopreventive intervention? We maintain that it is. Firstly, the epidemiological evidence that regular use of NSAIDs, such as aspirin, reduces cancer risk in a number of organs, especially the colorectum, is overwhelming, and it clearly supports the notion that cyclooxygenase inhibition has antineoplastic consequences (Thun *et al*, 2002). Secondly, prospective, randomised clinical trials using an intraepithelial neoplasia end-point suggest that two different NSAIDS, aspirin and sulindac, reduce recurrence of adenomatous polyps (Giardiello *et al*, 1993; Baron *et al*, 2003).

It is important to note that cyclooxygenase-independent mechanisms of action of NSAIDs may, at least in part, explain their anticancer activity (Thun *et al*, 2002). For example, the selective cyclooxygenase-2 inhibitor celecoxib has been demonstrated to inhibit AKT signalling and to induce apoptosis of human colorectal and prostate cancer cells *in vitro* in a cyclooxygenase-2-independent manner, by a mechanism involving direct inhibition of phosphoinositide-dependent kinase-1 (PDK-1) (Arico *et al*, 2002; Song *et al*, 2002). Whether this activity is relevant to the antineoplastic activity of celecoxib *in vivo* is unclear at present.

Currently, a number of clinical trials of the chemopreventive efficacy of the selective cyclooxygenase-2 inhibitors remain in analysis for colorectal adneocarcinoma, transitional cell carcinoma of the bladder, breast adenocarcinoma, cervical intra-epithelial neoplasia, lung cancer, skin cancer, and oral leukoplakia. While many of these trials have been disrupted following recognition of the cardiovascular toxicity of selective cyclooxygenase-2 inhibitors (Bresalier *et al*, 2005; Solomon *et al*, 2005), nevertheless, useful data will emerge from these studies, which will ultimately define the chemopreventive efficacy of these agents (Alberts *et al*, 2005). A more rigorous dose–response evaluation to ascertain the optimal dose and schedule required to delay or prevent cellular transformation may enable the use of lower doses that might not cause cardiovascular toxicity.

### Molecular targeted approaches other than cyclooxygenases

ErbB tyrosine kinases, the most prominent among them being epidermal growth factor receptor (EGFR), when phosphorylated activates signal transduction pathways that enhance cell survival, motility, and proliferation. Inhibitors of EGFR activation via antibodies to the extracellular protein or via tyrosine kinase inhibitory molecules provide promising new approaches to both cancer treatment and prevention. Despite mixed results in cancer treatment, many dysplastic cells also have activated EGFR and are reasonable targets for these agents. Preclinical data *in vitro* and *in vivo* support the potential preventive efficacy of these agents, potentially in combination with NSAIDs for chemoprevention of colorectal and head and neck cancers (Shin *et al*, 1994).

P53 targeting with ONYX-015, an adenovirus that replicates only in p53-deficient cells, produced histologic resolution of dysplasis in seven of 19 patients with oral leukoplakia (Rudin *et al*, 2003). Other p53-deficient dysplasias or high-risk epithelial fields might be protected with this agent.

Peroxisome proliferation activated receptor (PPAR) gamma agonists, such as rosiglitazone, are currently in early phase chemoprevention development. These agents are of particular interest for targeting hormone receptor negative breast epithelial proliferations or dysplasia (Mehta *et al*, 2000).

Hypermethylation contributes to carcinogenesis by silencing downstream transcription of key tumour suppressor genes. That aging and carcinogenesis are linked through progressive changes in methylation patterns of key tumour suppressor genes suggests potential future chemoprevention targets. Pharmacologic inhibition with well-tolerated interventions that inhibit or reduce activity of methyltransferase and histone deacetylase proteins may reduce abnormal hypermethylation of key tumour suppressor gene promoters, thus reducing or delaying neoplastic transformation. Hydralazine is one potentially useful DNA methylation inhibitor (Deng *et al*, 2003). A number of synthesised hydroxamic acid inhibitor of histone deacetylase inhibitors are currently in clinical trials for cancer treatment (Marks *et al*, 2000).

### Combined agents

The complexity and heterogeneity of known carcinogenesis mechanisms provides a strong rationale for combining drug interventions that might provide additive or synergistic anti-carcinogenesis effect. In addition to targeted anticarcinogenic mechanism-based approaches, population epidemiology and rodent models help in the choice of suitable agents for combinations. The most quoted example under investigation, the combination of inhibitors of EGFR signalling and cyclooxygenase (Torrance *et al*, 2000), which is currently under investigation in humans, may be modified due to the recent therapeutic index concerns for targeted cyclooxygenase inhibitors. Data *in vitro* and *in vivo* support the viability of combining HMG CoA reductase inhibitors and NSAIDs to increase apoptosis via enhanced caspase 3 activity in preneoplastic cells (Agarwal *et al*, 1999). DFMO and sulindac are currently in phase IIb clinical trial as a potential colorectal adenocarcinoma preventive combination (Gerner and Meyskens, 2004).

## PREVENTION OF SPORADIC EPITHELIAL NEOPLASIA IN POPULATIONS WITHOUT HIGH-RISK LESIONS

A population-based approach to chemopreventive intervention considers the gene–environmental risk of otherwise healthy populations. For example, individuals with high-frequency, low-penetrance polymorphisms such as the adenomatous polyposis coli II307K polymorphisms have a higher risk of developing colorectal adenocarcinoma, but this risk does not appear to become clinically apparent until later in life (Niell *et al*, 2003). Interventions or behaviour expected to reduce transformation risk have amplified protective effects in this cohort when compared to lower risk populations (Poynter *et al*, 2005).

One may consider the I1307K polymorphism as a paradigm for other single nucleotide polymorphisms associated with enhanced transformational risk. Otherwise healthy individuals who are found to carry such polymorphisms would reject high-cost, mildly toxic pharmaceutical interventions but might be receptive to dietary interventions or nutriceutical interventions.

### Nutriceuticals

Could there be ‘nontoxic alternatives’ to drugs with unacceptable therapeutic indicies such as NSAIDs or targeted therapeutic agents? Some recent results tentatively suggest that certain nutriceuticals may constitute such an alternative. Considerable evidence supports the hypothesis that flavonoids and other polyphenolic phytochemicals contained in certain foodstuffs, for example onions, red grapes, nuts and green tea, mediate, or contribute to, the putative cancer chemopreventive properties of their dietary sources. One potential approach is to deliver chemopreventive substances as a food supplement that is grown consistently between years to deliver constant concentrations of micronutrients. For example, black raspberries, which contain substantial concentrations of phenolic acids such as ellagic acid and ferulic acid, flavonoids such as anthocyanins, vitamins and minerals, may be grown with standardized content within and between years. When tested in rodent models, freeze-dried and frozen formulations of black raspberries inhibit colon tumours and aberrant crypt foci at concentrations of individual anticarcinogenesis constituents that are 10-fold less than the concentration of each individual constituent necessary to block aberrant crypt foci when given as a purified product (Harris *et al*, 2001). A whole food, given fresh or freeze dried as a capsule, containing multiple components thought to be anticarcinogenic is an efficient, nontoxic delivery system for potential anticarcinogenic compounds.

Alternatively, purified chemical isolates of foodstuffs may also be useful as preventive ‘drugs’. Such purified substances are commonly ingested in the food supply, sometimes in substantial quantities. Examples of such agents are curcumin, a constituent of curry, and resveratrol, contained in red grapes and berries. A plethora of mechanistic studies in cells *in vitro* suggests that polyphenolic phytochemicals can undermine oncogenic signalling cascades germane to tumour promotion and progression in a variety of ways (Manson *et al*, 2005). Both curcumin and resveratrol interfere with the cyclooxygenase-catalysed production of prostanoids from arachidonic acid by downregulation of arachidonic system catabolic enzymes (cyclooxygenases and lipoxygenases) and can reduce cancers in multiple rodent model systems of epithelial tumours (Leu and Maa, 2002; Aggarwal *et al*, 2004).

In the light of issue 2 outlined in the introduction, it seems most important to find out whether the potency difference in rodent colonic carcinogenesis models between NSAIDs and polyphenolic phytochemicals (Gescher, 2004) translates into a similar difference in the ability to achieve adenoma regression in humans. Alternatively, it is conceivable that efficacy in rodent models just ‘signals’ potential for activity in humans without potency implications, implying that a positive result for an intervention in the preclinical model renders its preliminary investigation in humans definitely worthwhile.

### Diet modification

Diets rich in fruits and vegetables, specifically diets rich in citrus fruits, dark green vegetables, and cruciferous vegetables, reduce risk of cancer. Recent data document success in diet modification using extensive prepared materials, staff effort, intensive intervention with the targeted population over a 1-year (Resnicow *et al*, 2001; Pierce *et al*, 2004) or 4-year period (Schatzkin *et al*, 2000). Diet can be modified in medically well-served (Schatzkin *et al*, 2000; Resnicow *et al*, 2001; Pierce *et al*, 2004) and medically underserved populations (Resnicow *et al*, 2001). The entire diet can be modified to enhance fruit and vegetable intake (Schatzkin *et al*, 2000; Resnicow *et al*, 2001; Pierce *et al*, 2004). Dietary interventions utilising motivational interviewing performed by minimally trained lay members of a community can successfully disseminate cancer protective diets to large populations at minimal cost (Resnicow *et al*, 2004).

A prospective, 4-year diet modification trial failed to reduce adenoma recurrence (Schatzkin *et al*, 2000). Similarly, an intervention with fibre also failed to reduce colorectal adenoma recurrence (Alberts *et al*, 2000) The former trial was criticised because the serum biomarker of diet modification, serum carotenoids, did not change in the treated group while the dose and type of fibre used was criticised in the later trial. The ongoing WHEL project (Pierce *et al*, 2004) tests the question of dietary modulation impact upon cancer preventive and treatment efficacy in a population of women with early-stage breast cancer (Pierce *et al*, 2004). The tools and procedures necessary to effect a dietary change in a large population (thousands) are available at reasonable cost. While attempts to modify human carcinogenesis with diet modification to date have been disappointing, the effects of these interventions may require much longer term follow-up (beyond 4 years). Alternatively, intervention prior to the appearance of a late carcinogenesis neoplastic event, such as a dysplastic lesion, in individuals with low-penetrance, high-frequency genetic haplotypes predictive for increased risk of an epithelial cancer may be required to demonstrate cancer preventive efficacy. Given the low toxicity, low cost, and relative ease of implementation, diet modification should be considered as a preferred preventive intervention in the healthy but a higher than normal risk population.

## SUMMARY

Recent publications have identified major weaknesses in the developmental strategies of cancer chemopreventive agents that have resulted in negative trial outcomes. Pivotal issues have included (1) concerns over the concept of acceptable therapeutic index for interventions in healthy populations; (2) the necessary preliminary data set required to support the development and implementation of research in humans; (3) the design and progression of research in humans aimed at defining preventive efficacy; and (4) the discovery and validation of biomarkers as potential surrogates for preventive intervention efficacy. Addressing these issues requires reorientation of the selection process of individuals at risk for a future cancer. Individuals at high risk for future transformation are more likely to accept the toxicity risks and financial costs of treatment-related toxicity in exchange for delay of cancer occurrence. Individuals without a known tissue or molecular high-risk factor for cancer will not tolerate toxicity that reduces quality of life.

To address these two groups, which represent opposite poles of a risk continuum, development of multiagent pharmacologic interventions for high-risk individuals is warranted. Future combinations of pharmaceutical cancer preventives might consist of agents targeted to specific signal transduction pathways associated with specific epithelial cellular targets. Diet modification, and food or nutritional extracts (‘nutriceuticals’) with known anticarcinogenesis activity may be more acceptable to healthy populations with known genetic risk based upon gene–environment interactions.
